# Comparing Sociodemographic Factors Associated with Disability between Immigrants and the Chilean-Born: Are There Different Stories to Tell?

**DOI:** 10.3390/ijerph9124403

**Published:** 2012-12-04

**Authors:** Baltica Cabieses, Kate E. Pickett, Helena Tunstall

**Affiliations:** 1 Universidad del Desarrollo, Avenida Las Condes 12.348 Lo Barnechea Santiago 7710162, Chile; 2 Department of Health Sciences, University of York, York YO10 5DD, England; E-Mail: kate.pickett@york.ac.uk; 3 Department of Geography, University of Edinburgh, Drummond Street, Edinburgh EH8 9XP, Scotland; E-Mail: htunsta2@staffmail.ed.ac.uk

**Keywords:** migrant population, disability, population-based study, social epidemiology, Latin America

## Abstract

This study explored a range of sociodemographic factors associated with disability among international immigrants in Chile, and compared them to the Chilean-born. Secondary data analysis of the Chilean population-based survey CASEN-2006 was conducted (268,873 participants). Main health outcomes: any disability and six different types of disability: visual, hearing, learning, physical, psychiatric and speaking (binary outcomes). Sociodemographic variables: Demographic factors (age, sex, marital status, urban/rural, ethnicity), socioeconomic status (SES: income, education, employment status, and an integrated indicator combining the SES measures through cluster analysis for the immigrant population), material factors (overcrowding, sanitation, housing quality) and migration related (country of origin and length of stay). Immigrants reported a significantly lower prevalence of any disability (3.55%), visual (1.00%) and physical disability (0.38%). Factors associated with any disability among immigrants were age, low SES or over 20 years duration of residence in Chile; while a range of sociodemographic factors were associated with disability in the Chilean-born. Conditional regression models by age group varied between populations, but SES remained significantly associated with disability across immigrants and the Chilean-born. However, there are no similar patterns of factors associated to different types of disability between the populations under study. Factors associated with disability varied between populations under study, but SES showed a consistent association with any disability in immigrants and the Chilean-born. Types of disability showed different patterns of factors associated to them between populations, which suggest the great complexity of underlying mechanisms related to disability in Chile.

## 1. Introduction

Disability is a complex multidimensional health outcome and has been defined as a worldwide public health priority [1,2]. More than one billion people around the world live with some form of disability and of them around 200 million suffer considerable difficulties in functioning. It is widely recognised that people with disabilities have poorer health outcomes, lower education achievements, less economic participation and higher rates of poverty than people without disabilities [2]. In addition, disability is closely linked to rapid demographic ageing of populations in many countries [3]. To address the complex concept of disability, the International Classification of Functioning, Disability and Health (ICF) has proposed a shift from a biomedical perspective to a broader social understanding of disability [4]. By including contextual factors in the classification, ICF allows the impact of the environment on a person’s functioning to be recorded [4,5].

Research on sociodemographic factors associated with disability has grown significantly in the past decade, including in the Latin American region [6,7,8,9,10,11,12]. This has highlighted the broad range of factors affecting disabled people, including age, gender, ethnicity, poverty, social exclusion, and stigma and discrimination [13,14,15,16]. Moreover, evidence from across the world indicates there is a complex relationship between disability and population health and quality of life, as disability is both a cause and a consequence of life course factors [17,18,19,20,21,22].

Little is known however about disability among migrant populations [23,24,25,26]. Movement of people within and between countries has become a central and necessary part of contemporary society, and migration has been recognised as an important determinant of global health and social development [27]. In opposition to internal migrants—people who move within their country—the focus of attention of this paper is particularly on international immigrants, that is people who reside in a different country from the one they were born. It is a result of a wide range of complex international and local socioeconomic and demographic mechanisms at different time points and across the life course. It ensures economic development for some, while it deepens social and economic vulnerability and ill-health for others [28,29]. After a selection process in which the healthiest people are more likely to migrate (the “healthy migrant effect”), some migrant populations have shown a rapid deterioration of their health when living in socioeconomic deprivation and vulnerability in the host country, which may also affect the health of their offspring [30,31,32,33].

Migration is a complex field. There are variations in the definitions, theories, and evidence on the relationship between migration and health over time, but the topic remains controversial and challenging. Migration is the process of moving from one place to another [34]. The United Nations (UN) defines an international migrant as a person who stays outside their usual country of residence for at least one year [35]. Attempts to define migration have been complex and diverse, involving a wide range of contexts, reasons and types of immigrant, from healthy migrants in search of better opportunities, to political refugees escaping civil wars in their countries of origin. Besides, the migration experience does not only imply the experience of *crossing frontiers*. There are also symbolic frontiers between one community and another, which are separated by cultural differences. Experiences of crossing each frontier—the geographical and the cultural—may occur at different times and may involve different meanings that are difficult to measure and interpret [36]. Several migration theories have been proposed in the past, and they are related to push and pull factors, demographic variations, globalization and international economic marked trade, individual decisional making processes, and other (see a narrative summary in [37]).

The characteristics of migration in Latin America have altered significantly over time reflecting changing economic and political circumstances [38]. The Economic Commission for Latin America and the Caribbean (ECLAC) has identified three major migration patterns in Latin America and the Caribbean: (i) Historical immigration into Latin America from overseas between the mid-nineteenth and mid-twentieth centuries, with a strong European component, (ii) Intra-regional migration, encouraged by socio-economic developments and structural factors, particularly during the period 1970–1990, and (iii) South-North migration flows during the last century, resulting in the loss of qualified workers in Latin America and the Caribbean, the development of the economic value associated with the remittances sent by migrants to their countries of origin and the emergence of immigrant communities [39,40]. A feminization of migration has also been observed in past decades, with higher rates of women from lower socioeconomic position migrating to other countries in the Latin America region to work in manual and domestic services [41,42]. There has been a growing South-South migration pattern within the Latin American region, resulting from movement of people living in relatively less developed countries to more developed neighbouring ones. Most of these immigrants come from other Latin American countries, mainly Peru, Argentina, Bolivia and Ecuador [43,44].

At the time this study was conducted, there were a limited number of studies assessing the relationship between migration and disability, but they suggested a significant burden of various types of disability among immigrant populations [45,46]. Disability has been associated with deterioration of work and labour opportunities, legal and employment contract status, socioeconomic conditions, access and use of healthcare, and the living conditions of immigrants’ children [23,24,25,26]. Given that some migrant populations already experience several challenges in the foreign country, such as lack of understanding of the cultural context [47,48] and the health care system [49], language barriers [50,51], stigma and discrimination [52], occupational hazards [53], social exclusion [54,55,56], and others, the interaction of these with the existence of disability might put immigrants and their families in a particularly vulnerable place that needs further policy attention. 

Chile is considered a middle-income country with an intermediate level of development [57,58]. Gross domestic product per capita reached $15,866 (USD) in 2010 [59]. It has a population of just over 16 million inhabitants, of whom 85% live in urban areas (and most of them—40%—in the Metropolitan Region of Santiago). Chile has experienced deep economic, demographic and geographical changes, including a progressive improvement of the health status of the population, a decline in the infant and general mortality rates, and an increase in life expectancy [58]. These significant improvements in the health status of the general population in Chile have been, to a large extent, a consequence of systematic public health policies developed during the last century [60,61]. However, not all socioeconomic groups have benefited from the described developments to the same extent [58]. There are significant differences in the health status of the Chilean populations when comparing the type of health system [57], geographical location [62], gender [63], and age [64], among others [65,66].

Some studies of disability in Chile have been completed, but none have focused on the international immigrant population [67,68,69]. These studies indicate that the prevalence of disability in Chile, at around 7%, is not as large as in other Latin American countries, but it contributes significantly to the global burden of disease and costs to the health care system. At the same time, immigration to Chile has rapidly increased in the past decade, reaching around 1.8% of the total population in 2007 [44]. Little is known about the health status of immigrants in the country and how this is related to different social determinants of health from a population-based analysis. Purposive sampled qualitative studies have indicated there is socioeconomic vulnerability and poor health among some groups of immigrants living in Chile [70,71]. Results from this study are the first to use survey analysis to assess the health of a representative sample of international immigrants in Chile.

This study aimed to analyse the prevalence of disability and factors related to disability among international immigrants in Chile, and compare them with the Chilean-born population. Three specific objectives were considered:

1. To determine the crude prevalence of any disability and different types of disability in the international immigrant population and compare them to the Chilean-born.
2. To analyse which demographic, socioeconomic, material living standards and migration-related factors are associated with any disability and different types of disability among immigrants and how they compare to the Chilean-born population.
3. To analyse which factors are associated with any disability and different types of disability among immigrants when stratified by age group (infant below 16 years old, working age between 16 and 65, and the elderly over 65 years old) and how they compare to the Chilean-born population.

## 2. Methods

### 2.1. Population and Sample

This study corresponds to a secondary data analysis of the 2006 CASEN (the acronym for Caracterización Socio-Económica Nacional) survey. The CASEN survey employed multistage probabilistic sampling, stratified by urban and rural area. The final sample for the analysis consisted of 268,873 people who belonged to a random sample of 73,720 households (44,854 urban and 28,866 rural ones), representing a 95.4% of the total Chilean territory [72,73]. The response rate of the 2006 CASEN survey was 84.8% [74]. Descriptive results of the sociodemographic characteristics of the total immigrant population and the Chilean-born have recently been published [75] and they indicate the CASEN survey is a close representation of the total immigrant population in Chile, as described by the Governmental Department of Migration in the past [44].

### 2.2. Migration Status

The 2006 CASEN survey asked the question: in which country was your mother living when you were born? Those who answered “in a different country from Chile” were identified as international immigrants, approximately 1% of the total sample (n = 1,877). An additional 0.7% preferred not to report their migration status and they were excluded from this analysis.

### 2.3. Self-Reported Disability

1. Any disability: dichotomous variable (yes/no) indicating the presence of one or more disabling conditions or no disabilities from 6 alternatives (visual, hearing, speaking, physical, cognitive, and psychiatric disability).
2. Six types of disability: all dichotomous variables (yes/no) indicating the presence of the following types of disability: visual, hearing, speaking, physical, cognitive, and psychiatric.
3. Causes of disability: nominal variable of four categories: birth, disease, accident, and other.

### 2.4. Demographic Factors

These include age, sex, marital status, urban versus rural area, area of the country, and belonging to any of the nine legally recognised pre-Hispanic ethnic groups living in the country.

### 2.5. Socioeconomic Status (SES)

1. Household income: household income per capita in the past month in Chilean pesos and converted to USD purchasing power parity for 2006 (PPP, continuous variable, USD$1 corresponds to 531 Chilean pesos in the 2006 currency) [59].
2. Educational level: the highest level achieved for each member of the household (categorical variable): university, technical, high-school, primary school or no education.
3. Employment status: binary variable indicating whether the head of the household reports having a job at the time of the interview (yes/no).
4. SES clusters among immigrants: given the great heterogeneity found in the SES of international immigrants in a previous study [75], we decided to include three ordinal categories of SES in this particular group: low, medium and high SES clusters. These categories emerged from hierarchical cluster analysis described elsewhere [75] and combined income, educational level and employment status of the international immigrant population.

### 2.6. Material Living Standards

Household material living conditions were measured using three self-reported variables as recommended by the Chilean Ministry of Planning [74]:

1. Housing quality: was measured by an index that included the quality of the walls, floor and ceiling. Housing quality was then categorised as high (constructed of solid materials), regular (poor quality but all enduring materials) and poor (constructed of one or more non-enduring materials, such as plastic or cardboard).
2. Sanitary systems: an adequate sanitary system, irrespective of urban/rural location, was defined as “0”, and a deficient sanitary system was labelled as “1”.
3. Household assets index (HAI): continuous variable obtained from the combination of nine household assets through principal component analysis (PCA), which accounted for 48% of the total variance [76,77]. This index ranges between −1.00 and 9.87. The nine assets were: car, washing machine, fridge, water heater, land phone, cable TV connection, computer, internet access, and mobile. Cronbach’s alpha test for internal reliability was adequate (0.81) and Kaiser-Meyer-Olkin measure of sampling adequacy was above the minimum recommended of 0.6 (0.88) [77].
4. Overcrowding, Townsend criteria: this was measured as indicated by the Townsend score of deprivation [78]. The variable is value 0 if the ratio is below 1.0 and obtains a value of 1 whenever this ratio is above 1.0 (more than 1 person per room in the household).

### 2.7. Migration-Related Factors

Country of origin (multinomial variable of five categories: Peru, Argentina, Ecuador, Bolivia and other) and years living in the country (continuous variable, range 0–62, and then divided into six categories: less than a year, 1–5 years, 6–10 years, 11–15 years, 16–20 years, and over 20 years). 

### 2.8. Analysis

Descriptive statistics for each self-reported health outcome under study are reported as means for continuous variables and proportions for categorical variables. Prevalence of each health outcome, crude and stratified by age groups, is also reported and includes parametric test of hypothesis by using chi-square and *t*-tests for independent samples.

The association between any disability and each type of disability, and demographic, socioeconomic, material and migration-related factors were analysed separately by logistic regression models that estimated crude and adjusted odds ratios (OR) with their 95% confidence intervals (robust standard errors method applied) [79,80]. These models estimated the crude probability of presenting any disability in the total population (unconditional model) and separated models by age group (conditional models) among the immigrant and compared to the Chilean-born population. All models were adjusted by a set of social determinants of health [81], age, sex, and zone rural or urban.

With regard to post-estimation tests of the regression models [82,83,84], the Archer and Lemeshow goodness of fit test for a logistic regression model fitted using survey sample data was estimated (F-adjusted mean residual GOF test) [85]. Regarding multiple comparison tests, the main risk is that there is a higher probability of finding differences between groups by chance (*i.e.*, false positives) [86,87,88]. For situations like the one presented in this exploratory study, it has been suggested that analyses should be made without multiple test adjustments and used instead in future confirmatory studies [89]. Therefore, no testing of multiple comparison or adjustment was conducted.

Data analyses were conducted with the STATA 11.0 statistical software package and estimations were weighted to take into account the complex multistage sampling strategy of the survey and to attain population-based estimations [90]. More detailed information of the methods considered in this study can be found in the on-line version of this paper.

## 3. Results

A description of population samples appears in Table 1. Further socioeconomic conditions of immigrants and how they compare to the Chilean-born population have been explored elsewhere [75]. The international immigrant population reported a significantly lower prevalence of any disability (3.55%, 95%CI 2.49–5.02) than the Chilean-born (6.93%, 95%CI 6.74–7.13) (see Table 2). This predominantly reflects the lower prevalence of both visual (1.00%, 95%CI 0.48–2.07) and physical (0.38%, 95%CI 0.19–0.76) disability types compared to the host population. Regarding causes of disability, immigrants reported a significantly lower prevalence of an accident (2.92%, 95%CI 0.99–8.26) than the Chilean-born (11.08%, 95%CI 10.29–18.29) and a significantly higher prevalence of “other” not stated cause (26.73%, 95%CI 13.00–47.11, versus 2.43%, 95%CI 2.00–2.95 among the Chilean-born).

**Table 1:** Sociodemographic characteristics of the Chilean-born and the international immigrant population in Chile, CASEN survey 2006 (weighted sample size 16,130,743 and 154,431, respectively).

	**Chilean-born population**		**International immigrant population**	
	**% or mean**	**95%CI**	**% or mean**	**95%CI**
**Sex (male)^ b^**	48.66	48.40–48.94	45.21	41.74–48.72
**Mean age**	*X* = 32.97	32.81–33.12	*X* = 33.41	31.81–35.00
**Age categories^ a^:**				
<16 ^c^	25.27	24.98–25.55	13.60	11.29–16.28
16–65 ^c^	66.41	66.12–66.70	79.08	75.92–81.93
Over 65	8.32	8.13–8.52	7.32	5.33–9.97
**Marital status^ a^:**				
Single ^b^	50.57	50.31–50.84	45.81	42.06–49.62
Married or cohabitant couple ^b^	40.76	40.46–41.06	45.49	41.66–49.36
Annulled, separated or divorced	4.56	4.42–4.71	4.21	3.06–5.77
Widow	4.07	3.95–4.19	4.49	2.89–6.91
**Minority ethnic group: any**	6.55	6.52–6.80	5.57	3.79–8.10
**Zone: Rural^ a^**	12.86	12.59–13.14	6.03	4.89–7.42
**Area^ a^:**				
Northern	11.80	11.58–12.03	13.15	10.14–16.89
Central ^b^	62.06	61.76–62.36	73.66	69.22–77.66
Southern ^b^	26.14	25.90–26.37	13.19	10.50–16.45
**Educational level^ a^:**				
No education ^b^	7.39	7.23–7.55	2.38	1.51–3.73
Primary School ^b^	34.68	34.33–35.03	18.79	16.05–21.88
High School	29.68	29.34–30.03	33.02	29.39–36.87
Technical level	14.51	14.24–14.79	16.81	14.13–19.88
University level ^b^	9.86	9.57–10.15	27.32	23.16–31.98
**Mean household income, per capita ($USD)^ a^:**				
Quintile 1 (poorest)	58.57	57.88–59.26	56.78	50.81–62.74
Quintile 2	107.98	107.55–108.41	110.03	106.51–113.54
Quintile 3	159.22	158.69–159.75	162.62	157.81–167.43
Quintile 4	243.23	242.18–244.28	245.37	238.25–252.50
Quintile 5 (wealthiest) ^c^	778.97	757.28–800.67	1,305.60	1,070.18–1,541.03
Current active worker (yes)	57.16	56.84–57.48	60.96	57.06–64.73

^a ^*p* < 0.0001 when comparing categories within the same variable for either the Chilean-born or the IIP; ^b ^*p* < 0.05 when comparing the same category across populations, the Chilean-born population versus the international immigrant population; ^c ^*p* < 0.0001 when comparing the same category across populations, the Chilean-born population versus the international immigrant population.

**Table 2:** Prevalence of disability of the Chilean-born and the international immigrant population in Chile, CASEN survey 2006 (weighted sample size 16,130,743 and 154,431, respectively).

Dimensions		Chilean-born population		International immigrant population	
		%	95%CI	%	95%CI
**Any disability^ b^**		6.93	6.74–7.13	3.55	2.49–5.02
**Type of disability^ a^:**					
Visual ^b^		3.17	3.05–3.28	1.00	0.48–2.07
Hearing		1.22	1.16–1.29	0.59	0.22–1.58
Speaking		0.32	0.29–0.36	0.19	0.039–0.95
Physical ^b^		2.15	2.06–2.24	0.38	0.19–0.76
Learning		0.86	0.80–0.91	0.23	0.074–0.74
Psychiatric		0.41	0.36–0.45	0.21	0.059–0.71
**Cause of disability^ a^:**					
Birth		23.65	22.45–24.89	23.09	10.64–43.06
Disease		45.66	44.29–47.04	45.15	28.70–62.73
Accident ^b^		11.08	10.29–18.29	2.92	0.99–8.26
Other ^b^		2.43	2.00–2.95	26.73	13.00–47.11
**Country of origin (immigrants only)**					
Peru		-	-	0.40	0.14–1.16
Argentina		-	-	0.75	0.47–1.20
Bolivia		-	-	0.23	0.08–0.64
Ecuador		-	-	0.18	0.05–0.58
Other		-	-	1.95	1.12–3.39
**Years living in the country (immigrants only)**					
Less than a year		-	-	0.80	0.41–1.53
1 to 5 years		-	-	0.25	0.05–1.10
6 to 10 years		-	-	0.56	0.20–1.56
11 to 15 years		-	-	0.06	0.001–0.27
16 to 20 years		-	-	0.02	0.006–0.11
21 or more years		-	-	1.85	1.12–3.03

^a^ *p* < 0.0001 when comparing categories within the same variable for either population; ^b^ *p* < 0.0001 when comparing the Chilean-born population with the international immigrant population.

In relation to migration-related factors among immigrants, the highest prevalence of any disability was found within the “other types” of countries of origin (1.95%) followed by Argentina (0.75%) and Peru (0.40%). The lowest rate was found among immigrants coming from Ecuador. Possibly due to age, the highest rate of any disability was found among immigrants with over 20 years of length of stay in the country (1.85%), but no clear gradient of any disability by years living in Chile was observed (see Table 2).

### 3.1. Factors Associated with Any Disability: Comparisons of Partially Adjusted Models between the Immigrant and Chilean-Born Populations

Among immigrants, the only significant factors associated with any disability were age (OR 1.04, 95%CI 1.02–1.06), Low SES cluster (OR 3.21, 95%CI 1.21–8.48, trend *p* > 0.05), and duration of residence over 20 years (OR 2.95, 95%CI 1.09–8.00, trend *p* > 0.05). Among the Chilean-born in contrast, a range of factors were associated with the chance of being disabled, including the cause of disability, demographic and socioeconomic conditions, and material living standards. In addition, a multiplicative protective interaction term was found between overcrowding and age (being older and living in an overcrowded household reduced by 0.01% the chance of being disabled by every year of age); and between educational level and income (the higher the educational level and the income the lower the likelihood of being disabled by 0.03% per unit of increase in each category) (Table 3).

**Table 3:** Adjusted Odds Ratio (OR)^α^ of presenting *any disability* in Chile, a comparison between the Chilean-born and the international immigrant Population, CASEN-2006 survey (weighted sample size 16,130,743 and 154,431, respectively) (statistical significant trends appear in grey shade in the table).

Social Determinants	Chilean-Born Population		International Immigrant population	
	OR	95%CI	OR	95%CI
**CAUSE OF DISABILITY**				
Birth	**1.00**	**-**	1.00	-
Disease	1.48 **	**1.22**–**1.79**	0.81	0.04–13.73
Accident	0.71 *	**0.53**–**0.95**	3.70	0.17–8.59
Other	2.37 **	**1.90**–**2.94**	0.72	0.02–4.84
***GOF test***	***p*** > ***0.05*** (***adequate***)		***p*** > ***0.05*** (***adequate***)	
**DEMOGRAPHICS**				
**Age**	**1.05** **	**1.04**–**1.05**	**1.04** **	**1.02**–**1.06**
**Sex (female** ** =** ** 1)**	0.94	0.89–1.004	0.56	0.25–1.25
**Marital status:**				
Single	**1.00**	**-**	1.00	-
Married	0.47 **	**0.44**–**0.51**	0.79	0.29–2.17
Divorced	0.69 **	**0.61**–**0.78**	2.57	0.52–12.73
Widow	0.62 **	**0.56**–**0.69**	1.07	0.26–4.39
**Ethnicity: any**	0.71	0.35–1.44	1.06	0.17–6.48
**Zone (Rural** ** =** ** 1)**	0.80 **	**0.75–0.85**	1.56	0.80–3.04
**Area:**				
Northern	1.00	-	1.00	-
Central	1.00	0.90–1.12	0.48	0.14–1.64
Southern	1.02	0.91–1.14	0.89	0.27–2.91
***GOF test***	***p*** > ***0.05*** (***adequate***)		***p*** > ***0.05*** (***adequate***)	
**SOCIOECONOMIC FACTORS**				
**Educational level:**				
No education	3.70 **	**3.16**–**4.32**	1.94	0.41–9.12
Primary School	2.50 **	**2.17**–**2.88**	1.95	0.70–5.40
High School	1.52 **	**1.31**–**1.75**	1.05	0.37–2.91
Technical level	1.24 **	**1.05**–**1.47**	0.07	0.01–0.48
University level	**1.00**	**-**	1.00	-
**Household income, per capita:**				
Quintile 1 (poorest)	2.58 **	**2.34**–**2.85**	2.09	0.85–5.10
Quintile 2	1.87 **	**1.69**–**2.08**	1.53	0.57–4.13
Quintile 3	1.60 **	**1.44**–**1.79**	0.68	0.18–2.51
Quintile 4	1.28 **	**1.14**–**1.43**	1.14	0.33–3.92
Quintile 5 (wealthiest)	**1.00**	**-**	1.00	-
Current worker	**0.38** **	**0.27**–**0.53**	4.31	0.43–9.63
Interaction: Overcrowding Townsend score * Age	**0.99** **	**0.99**–**0.99**	-	-
Interaction: Educational level * Household income	**0.97** *	**0.95**–**0.99**	-	-
***GOF test***	***p***> ***0.05***(***adequate***)		***p***> ***0.05***(***adequate***)	
**SOCIOECONOMIC CLUSTERS**				
Low SES cluster	**-**	**-**	**3.21** **	**1.21**–**8.48**
Medium SES cluster	**-**	**-**	1.65	0.66–4.15
High SES cluster	**-**	**-**	1.00	-
***GOF test***	***N/A***		***p***> ***0.05***(***adequate***)	
**MATERIAL FACTORS**				
**Quality of the household Index:**				
Acceptable	**1.00**	**-**	1.00	-
Sub-standard	1.26 **	**1.18**–**1.34**	0.90	0.44–1.81
Unfit	1.90 **	**1.54**–**2.35**	4.37	0.86–22.01
Sanitary Index (deficient = 0)	1.04	0.98–1.10	0.82	0.37–1.81
Overcrowded household (Townsend):	**0.69** **	**0.64**–**0.74**	0.58	0.26–1.30
HAI	**0.96** **	**0.94**–**0.98**	0.94	0.87–1.07
***GOF test***	***p***> ***0.05***(***adequate***)		***p***> ***0.05***(***adequate***)	
**MIGRATION RELATED FACTORS**				
**Years living in the country:**				
Less than a year	-	-	1.00	-
1 to 5 years	-	-	0.76	0.14–4.09
6 to 10 years	-	-	1.72	0.48–6.17
11 to 15 years	-	-	0.37	0.06–2.64
16 to 20 years	-	-	0.13	0.02–1.65
21 or more years	-	-	**2.95** **	**1.09**–**8.00**
**Country of origin:**				
Peru	-	-	0.49	0.13–1.78
Argentina	-	-	0.58	0.25–1.36
Bolivia	-	-	0.85	0.24–3.01
Ecuador	-	-	1.38	0.27–6.95
***GOF test***	***N/A***		***p*** > ***0.05***(***adequate***)	

^α ^Models adjusted by age, sex, urban/rural; Archer and Lemeshow GOF test for weighted logistic regressions displayed for each model; * *p* < 0.05, ** *p* < 0.001.

### 3.2. Comparing Factors Associated with Any Disability by Populations’ Age Groups

Table 4 displays the factors associated with any disability among immigrants by age groups, those being children (below 16 years old), working age (16 to 65 years old) and the elderly (over 65 years old). The child population indicated a significant association between any disability and being female (OR 0.13, 95%CI 0.02–0.78), area of the country (lower in the northern area, trend *p* < 0.05), and SES cluster, with a clear negative gradient (trend *p* < 0.05). Among the adult immigrant group, age (OR 1.06, 95%CI 1.02–1.10) and being employed (OR 3.93, 95%CI 1.08–15.45) were associated with any disability, whereas among the immigrant elderly population, age (OR 1.33, 95%CI 1.12–1.56), being widowed (OR 6.23, trend *p* > 0.05), and SES cluster (clear negative gradient, trend *p* < 0.05) were associated with this health outcome (see Table 3). The Chilean-born populations also reported significant associations with these variables and, in addition, ethnicity, living in rural areas, marital status and various material living standards (see Table 5). No multiplicative terms were found in these analyses. 

**Table 4:** Adjusted Odds Ratio (OR)^α^ of presenting any disability in the international immigrant population by age groups, CASEN survey, 2006 (weighted sample size 154,431)(statistical significant values appear in grey shade in the table).

Variables	Immigrants under 16 years old		Working age immigrants (16 to 65)		Elderly immigrants (over 65)	
	OR	95%CI	OR	95%CI	OR	95%CI
**CAUSES OF DISABILITY**						
Birth	1.00	-	1.00	-	1.00	-
Disease	0.94	0.05–3.90	1.18	0.76–8.37	3.42	0.78–12.43
Accident	3.46	0.98–16.6	1.98	0.88–5.18	1.24	0.16–8.12
Other	0.88	0.20–3.50	0.98	0.04–5.32	2.61	0.45–9.76
***GOF test***	***p*** > ***0.05***(***adequate***)		***p*** > ***0.05*** (***adequate***)		***p*** > ***0.05***(***adequate***)	
**DEMOGRAPHICS**						
**Age**	0.92	0.81–1.05	**1.06** *	**1.02**–**1.10**	**1.33** **	**1.12**–**1.56**
**Sex (female** ** =** ** 1)**	**0.13** **	**0.02**–**0.78**	0.61	0.20–1.77	1.02	0.28–3.62
**Marital status:**						
Single	-	-	1.00	-	1.00	-
Married	-	-	0.54	0.15–1.85	**19.44** **	**1.44**–**23.74**
Divorced	-	-	1.79	0.28–11.39	-	-
Widow	-	-	1.19	0.14–10.01	4.31	0.45–15.23
**Ethnicity: any**	0	-	0.49	0.06–3.88	**6.23** **	**2.35**–**13.43**
**Zone: rural** ** =** ** 1**	1.19	0.19–7.26	1.02	0.32–3.29	3.13	0.64–15.13
**Area:**						
Northern	**1.00**	**-**	1.00	-	1.00	-
Central	8.77 **	**1.11**–**16.88**	0.40	0.09–1.69	0.17	0.01–1.82
Southern	3.08 **	**1.99**–**4.76**	0.50	0.12–2.07	2.98	0.41–21.42
***GOF test***	***p*** > ***0.05***(***adequate***)		***p*** > ***0.05***(***adequate***)		***p*** > ***0.05***(***adequate***)	
**SOCIOECONOMIC FACTORS**						
**Educational level:**						
No education	-	-	2.64	0.26–26.77	6.31	0.42–92.53
Primary School	-	-	1.08	0.16–7.23	9.50	1.15–78.07
High School	-	-	1.08	0.20–5.80	4.28	0.43–41.23
Technical level	-	-	-	-	-	-
University level	-	-	1.00	-	1.00	-
**Household income:**						
Quintile 1 (poorest)	-	-	3.10	0.56–17.09	2.58	0.45–14.61
Quintile 2	1.76	0.12–25.42	0.23	0.01–2.84	3.34	0.48–39.54
Quintile 3	0.46	0.02–7.65	1.44	0.1 6–12.72	0.03	0.001–0.74
Quintile 4	1.45	0.09–21.25	2.42	0.2 8–20.39	1.09	0.19–9.96
Quintile 5 (wealthiest)	1.00	-	1.00	-	1.00	-
Current worker	-	-	**3.93** *	**1.08**–**15.45**	-	-
***GOF test***	***p*** < ***0.05***(***poor***)		***p*** > ***0.05*** (***adequate***)		***p*** > ***0.05***(***adequate***)	
**SOCIOECONOMIC CLUSTERS**						
Low SES cluster	**8.37** *	**1.03**–**16.79**	3.16	1.09–9.16	**3.46** *	**1.74**–**20.31**
Medium SES cluster	**5.03** **	**3.02**–**8.32**	1.24	0.44–3.44	6.37	0.96–42.10
High SES cluster	**1.00**	**-**	1.00	-	**1.00**	**-**
***GOF test***	***p*** > ***0.05*** (***adequate***)		***p*** > ***0.05*** (***adequate***)		***p*** >***0.05***(***adequate***)	
**MATERIAL FACTORS**						
**Quality of the household:**						
Acceptable	1.00	-	1.00	-	1.00	-
Sub-standard	0.29	0.01–6.07	0.33	0.01–1.33	7.79	0.01–15.35
Unfit	-	-	3.96	0.03–9.66	3.97	0.01–11.38
Sanitary Index (deficient = 0)	28.34	0.79–1.57	1.14	0.00–3.35	0.06	0.03–10.59
**Overcrowding (Townsend):**	0.10	0.01–7.16	0.30	0.04–2.11	11.62	0.07–18.88
HAI	-	-	8.00	0.02–13.99	0.3	0.06–1.32
CMI	0.01	0.001–13.98	0.1	0.01–1.23	4.65	0.07–12.88
***GOF test***	***p*** < ***0.05*** (***poor***)		***p*** < ***0.05***(***poor***)		***p*** < ***0.05***(***poor***)	
**MIGRATION RELATED FACTORS**						
**Years living in the country:**						
Less than a year	1.00	-	1.00	-	1.00	-
1 to 5 years	-	-	1.17	0.21–6.25	-	-
6 to 10 years	-	-	1.57	0.34–7.22	0.4	0.03–3.26
11 to 15 years	-	-	0.39	0.05–8.23	2.27	0.07–6.55
16 to 20 years	-	-	0.14	0.02–0.98	-	-
21 or more years	-	-	3.09	0.81–11.78	-	-
**Country of origin:**						
Peru	1.04	0.08–13.16	0.31	0.06–1.58	13.98	0.60–32.01
Argentina	0.34	0.04–3.81	0.41	0.15–1.13	1.60	0.18–14.16
Bolivia	-	-	0.37	0.09–1.41	-	-
Ecuador	-	-	1.33	0.26–6.81	-	-
***GOF test***	***p*** < ***0.05***(***poor***)		***p*** < ***0.05*** (***poor***)		***p*** < ***0.05***(***poor***)	

^α ^Models adjusted by age, sex, urban/rural; Archer and Lemeshow GOF test for weighted logistic regressions displayed for each model; * *p* < 0.05; ** *p* < 0.001.

**Table 5:** Adjusted Odds Ratio (OR)^α^ of presenting any disability in the Chilean-born population by age groups, adjusted by socio-demographics, CASEN survey, 2006 (weighted sample size 16,130,746) (statistical significant values appear in grey shade in the table).

Variables	Under 16 years old		Working age (16 to 65)		Elderly (over 65)	
	OR	95%CI	OR	95%CI	OR	95%CI
**CAUSES OF DISABILITY**						
Birth	1.00	-	1.00	-	1.00	-
Disease	1.57 **	**1.31**–**1.91**	1.47 **	**1.21**–**1.79**	1.17	0.81–1.77
Accident	0.78	0.54–1.95	0.72	0.54–0.95	1.12	0.84–1.55
Other	1.35	0.89–1.92	2.31	1.29–2.94	0.85	0.79–1.32
***GOF test***	***p*** > ***0.05***(***adequate***)		***p*** > ***0.05***(***adequate***)		***p*** > ***0.05***(***adequate***)	
**TDEMOGRAPHICS**						
**Age**	**1.08** **	**1.06**–**1.10**	**1.05** **	**1.04**–**1.06**	**1.05** **	**1.04**–**1.06**
**Sex (female** ** =** ** 1)**	0.89	0.74–1.07	**0.73** **	**0.67**–**0.79**	0.99	0.90–1.10
**Marital status:**						
Single	1.00	-	**1.00**	**-**	1.00	-
Married	-	-	0.43 **	**0.39**–**0.47**	**0.78** *	**0.65**–**0.93**
Divorced	-	-	0.66 **	**0.57**–**0.76**	0.83	0.64–1.02
Widow	-	-	0.55 **	**0.46**–**0.66**	2.30	0.66–7.92
**Ethnicity: any**	**1.46** **	**1.11**–**1.91**	**1.18** *	**1.03**–**1.36**	1.07	0.89–1.30
**Zone: rural** ** =** ** 1**	**0.77** *	**0.65**–**0.91**	**0.75** *	**0.70**–**0.82**	**0.88** *	**0.79**–**0.97**
**Area:**						
Northern	1.00	-	1.00	-	1.00	-
Central	1.03	0.76–1.40	1.06	0.93–1.21	0.84	0.61–1.01
Southern	0.94	0.67–1.28	1.08	0.95–1.23	0.86	0.71–1.04
***GOF test***	***p*** > ***0.05***(***adequate***)		***p*** > ***0.05***(***adequate***)		***p*** > ***0.05***(***adequate***)	
**SOCIOECONOMIC FACTORS**						
**Educational level:**						
No education	-	-	2.08 **	**1.58**–**3.59**	1.52	1.08–1.95
Primary School	-	-	1.68 **	**1.22**–**2.31**	1.52	1.10–2.09
High School	-	-	1.33	0.98–1.79	1.32	0.95–1.83
Technical level	-	-	1.32	0.97–1.80	0.88	0.46–1.69
University level	-	-	1.00	-	1.00	-
**Household income:**						
Quintile 1 (poorest)	2.09 **	**1.52**–**2.88**	**1.45** **	**1.25**–**1.68**	**1.47** **	**1.19**–**1.82**
Quintile 2	1.84 **	**1.39**–**2.45**	**1.35** **	**1.18**–**1.56**	**1.44** **	**1.19**–**1.73**
Quintile 3	1.73 **	**1.26**–**2.38**	**1.35** *	**1.18**–**1.55**	**1.39** *	**1.15**–**1.66**
Quintile 4	1.24	0.90–1.70	**1.24** *	**1.08**–**1.43**	**1.36** *	**1.13**–**1.63**
Quintile 5 (wealthiest)	1.00	-	1.00	-	**1.00**	**-**
Currently worker	-	-	**0.75** *	**0.63**–**0.88**	-	-
***GOF test***	***p*** < ***0.05***(***not adequate***)		***p*** > ***0.05*** (***adequate***)		***p*** > ***0.05***(***adequate***)	
**MATERIAL FACTORS**						
**Quality of the household:**						
Acceptable	**1.00**	**-**	**1.00**	**-**	**1.00**	**-**
Sub-standard	2.50 **	**1.89**–**3.31**	0.10 **	**0.08**–**0.48**	0.17 **	**0.05**–**0.96**
Unfit	7.05 **	**3.40**–**11.46**	0.40 *	**0.01**–**0.90**	0.30 *	**0.02**–**0.95**
Sanitary Index (deficient = 0)	1.10	0.81–1.41	**2.54** **	**1.86**–**7.55**	**12.92** **	**2.57**–**64.52**
Overcrowded household (Townsend):	**2.26** **	**1.62**–**3.13**	**0.10** **	**0.01**–**0.03**	**0.70** *	**0.01**–**0.91**
HAI	-	-	**2.18** **	**1.18**–**4.02**	**8.74** **	**3.67**–**20.08**
***GOF test***	***p*** < ***0.05***(***not adequate***)		***p*** < ***0.05***(***not adequate***)		***p*** < ***0.05***(***not adequate***)	

^α ^Models adjusted by age, sex, urban/rural; Archer and Lemeshow GOF test for weighted logistic regressions displayed for each model; * *p* <0.05; ** *p* < 0.001.

### 3.3. Comparing Different Types of Disability between the International Immigrant and the Chilean-Born Populations

Factors associated with each type of disability were explored in both populations under study (Table 6 and Table 7). Immigrants with *visual disability* were more likely to live in central (OR 4.06, 95%CI 1.10–14.95) and southern areas (OR 9.89, 95%CI 2.35–41.66), to live in overcrowded households (OR 2.14, 95%CI 1.13–4.07) and to live with deficient sanitary conditions (OR 0.03, 95%CI 0.01–0.90). They were also less likely to come from Bolivia (OR 0.22, 95%CI 0.05–0.99). The Chilean-born with visual disability reported a significant association with cause of disability, age, sex, ethnicity and living in rural areas.

Regarding *hearing disability*, coming from Argentina was the only significant factor associated with disability among immigrants (OR 0.08, 95%CI 0.01–0.80), whereas age, sex, marital status and being employed were factors significantly associated with this disability type in the Chilean-born. Living in an overcrowded household was the only variable associated with *speaking disability* among immigrants (OR 0.09, 95%CI 0.03–0.27), while educational level was the only factor associated in the Chilean-born (clear negative gradient, trend *p* < 0.05). 

Factors associated with *physical disability* in the international immigrant population were living in rural areas (OR 3.93, 95%CI 2.53–8.32), SES cluster (clear negative gradient, trend *p* < 0.05) and coming from Argentina (OR 9.55, 95%CI 1.29–27.74) and Peru (OR 3.98, 95%CI 1.29–8.32). In the Chilean-born in contrast, a broad range of variables were associated with physical disability including causes of disability and demographic characteristics. Immigrants with a *learning disability* were more likely to report a disease and an accident as the cause of this disability (OR 46.62 and 3.52, respectively) and to live in rural areas (OR 8.50, 95%CI 1.54–47.20). The Chilean-born with a *learning disability* were more likely to report birth defects, to be older, male, single, and to live in households with good material conditions. They were also less likely to be well educated. Finally, the only significant factors associated with *psychiatric disability* among international immigrants were age (OR 1.06, 95%CI 1.01–1.11) and coming from Ecuador (OR 4.35, 95%CI 1.65–8.90), while a range of factors were found to have a significant association with this type of disability among the Chilean-born. No multiplicative terms were found in these analyses. A summary table (Table 8) displaying the key factors associated with each type of disability can be found below.

**Table 6:** Adjusted Odds Ratio (OR)^α^ of presenting each type of disability in the international immigrant population, CASEN survey, 2006 (weighted sample size 154,431) (statistical significant values appear in grey shade in the table).

Variables	Visual		Hearing		Speaking		Physical		Learning		Psychiatric	
	OR	95%CI	OR	95%CI	OR	95%CI	OR	95%CI	OR	95%CI	OR	95%CI
**CAUSES OF DISABILITY**																									
Birth disability	1.00	-	1.00	-	1.00	-	1.00	-	1.00	-	1.00	-
Disease	0.84	0.05–13.9	3.00	0.07–11.3	3.00	0.07–11.30	2.18	0.26–18.3	46.62 **	**2.59**–**83.60**	0.05	0.005–6.34
Accident	3.76	0.18–76.6	0	-	0	-	1.48	0.08–25.1	3.52 **	**1.56**–**79.11**	0	-
Other	0.70	0.02–23.5	0	-	0	-	0	-			0	-
***GOF test***	***p*** > ***0.05***		***p*** < ***0.05***		***p*** < ***0.05***		***p*** > ***0.05***		***p*** < ***0.05***		***p*** < ***0.05***	
**DEMOGRAPHICS**																									
**Age **	1.01	0.99–1.04	1.01	0.97–1.06	1.02	0.98–1.07	1.04	0.99–1.10	0.93	0.82–1.06	**1.06** *	**1.01**–**1.11**
**Sex **(**female = 1**)	1.05	0.22–4.87	1.16	0.13–9.69	0	-	0.86	0.18–3.99	0.25	0.04–1.39	0.72	0.04–12.45
**Marital status: **												
Single	1.00	-	1.00	-	1.00	-	1.00	-	1.00	-	1.00	-
Married	0.96	0.08–1.37	3.12	0.22–43.3	0	-	0.38	0.10–1.43	0	-	0	-
Divorced	4.14	0.46–36.57	16.8	0.62–45.2	0	-	0.17	0.01–1.70	0	-	0	-
Widow	0	-	2.71	0.04–15.9	0	-	0.16	0.008–3.35	0	-	0	-
**Ethnicity: any **	0.34	0.08–1.37	1.03	0.06–16.6	0	-	1.17	0.22–6.18	0.19	0.01–1.93	0	-
**Zone: rural** ** =** ** 1**	0.96	0.46–36.57	1.52	0.43–5.32	0	-	**3.93** *	**1.31**–**11.7**	**8.50** **	**1.54**–**47.2**	3.19	0.08–36.92
**Area: **												
Northern	1.00	-	1.00	-	1.00	-	1.00	-	1.00	-	1.00	-
Central	4.06 *	**1.10**–**14.95**	0.26	0.01–4.65	0	-	1.40	0.29–6.70	0	-	0	-
Southern	9.89 **	**2.35**–**41.66**	0.14	0.08–2.40	0	-	1.20	0.18–7.73	0	-	0	-
***GOF test***	***p*** > ***0.05***		***p*** > ***0.05***		***p*** < ***0.05***		***p*** > ***0.05***		***p*** < ***0.05***		***p*** < ***0.05***	
**SOCIOECONOMIC FACTORS**																									
**Educational level: **												
No education	0	-	1.67	0.28–9.69	0	-	3.30	0.25–45.80	0	-	0	-
Primary School	0	-	0.27	0.05–1.47	0	-	8.11	0.87–15.9	0	-	0	-
High School	3.69	0.34–39.77	0.35	0.05–1.32	0	-	19.90	1.98–29.9	0	-	0	-
Technical level	4.63	0.56–38.08	-	-	0	-	1.65	0.08–31.25	0	-	0	-
University level	1.00	-	1.00	-	1.00	-	1.00	-	1.00	-	1.00	-
**Household income: **												
Quintile 1 (poorest)	2.68	0.06–11.23	1.17	0.16–8.45	0	-	3.40	0.45–25.19	0	-	0	-
Quintile 2	0.65	0.04–9.85	0.40	0.04–0.48	0	-	32.39	4.25–230.9	0	-	0	-
Quintile 3	0	-	0.60	0.06–0.66	0	-	0.50	0.05–0.95	0	-	0	-
Quintile 4	0	-	5.51	0.81–35.5	0	-	5.10	0.50–51.47	0	-	0	-
Quintile 5 (wealthiest)	1.00	-	1.00	-	1.00	-	1.00	-	1.00	-	1.00	-
Currently worker	2.01	0.29–13.91	0	-	0	-	0	-	0	-	0	-
***GOF test***	***p*** > ***0.05***		***p*** < ***0.05***		***p*** < ***0.05***		***p*** > ***0.05***		***p*** < ***0.05***		***p*** < ***0.05***	
**SES CLUSTERS**																									
Low SES cluster	3.29	0.71–7.72	0.68	0.09–5.12	-	-	5.18 **	**1.82**–**21.80**	-	-	-	--		
Medium SES cluster	0.70	0.17–2.89	0.84	0.10- 6.76	-	-	2.14 **	**1.74**–**11.95**	-	-	-			
High SES cluster	1.00	-	1.00		1.00	-	1.00	-	1.00	-	1.00	-
***GOF test***	***p*** > ***0.05***		***p*** > ***0.05***		***p*** < ***0.05***		***p*** > ***0.05***		***p*** < ***0.05***		***p*** < ***0.05***	
**MATERIAL FACTORS**																									
**Quality of the household: **												
Acceptable	1.00	-	1.00		1.00	-	1.00	-	1.00	-	1.00	-
Sub-standard	2.49	0.19–31.97	0.62	0.02–1.89	4.56	0.30–44.7	0.35	0.04–3.13	1.43	0.56–3.59	2.61	0.01–35.42
Unfit	8.11	0.01–19.90	9.68	0.01–6.35	0	-	0.03	0.01–0.94	0	-	0	-
Sanitary Index (deficient = 0)	**0.03** *	**0.01**–**0.90**	0.20	0.01–6.49	0	-	1.65	0.07–38.8	0.64	0.10–4.04	0.07	0.08–6.80
Overcrowded household (Townsend):	**2.14** *	**1.13**–**4.07**	0.36	0.01–8.57	**0.09** **	**0.03**–**0.27**	0.97	0.44–2.14	1.05	0.51–2.15	0.78	0.04–13.03
HAI	0.01	0.02–13.71	0.03	0.05–1.80	0	-	0.05	0.001–6.3	0.38	0.01–1.45	0.08	0.01–4.52
***GOF test***	***p*** > ***0.05***		***p*** < ***0.05***		***p*** < ***0.05***		***p*** > ***0.05***		***p*** > ***0.05***		***p*** > ***0.05***	
**MIGRATION RELATED FACTORS**																									
**Years living in Chile:**												
Less than a year	1.00	-	1.00	-	1.00	-	1.00		1.00	-	1.00	-
1 to 5 years	0.16	0.01–1.84	3.97	0.41–37.6	-	-	-	-	0.21	0.01–2.52	-	-
6 to 10 years	3.07	0.43–21.65	-	-	-	-	0.41	0.05–2.94	8.58	0.51–14.20	-	-
11 to 15 years	-	-	0.43	0.03–6.27	-	-	0.20	0.02–0.28	1.01	0.01–9.83	-	-
16 to 20 years	0.72	0.06–7.72	0	-	-	-	0.20	0.02–2.00	1.43	0.08–23.92	-	-
21 or more years	1.47	0.22–9.71	8.61	0.38–19.1	-	-	0.32	0.04–2.45	1.25	0.04–34.20	-	-
**Country of origin:**												
Peru	-	-	1.70	0.29–10.0	-	-	**3.98** **	**2.53**–**8.32**	-	-	5.38	0.96–44.95
Argentina	0.61	0.14–2.57	**0.08** *	**0.01**–**0.80**	-	-	**9.55** **	**1.29**–**27.74**	-	-	-	-
Bolivia	**0.22** *	**0.05**–**0.99**	0.63	0.07–5.38	-	-	4.80	0.75–31.09	-	-	-	-
Ecuador	0.92	0.07–11.12	1.78	0.10–31.3	-	-	-	-	-	-	**4.35** **	**1.65**–**8.90**
***GOF test***	***p*** < ***0.05***		***p*** < ***0.05***		***p*** < ***0.05***		***p*** > ***0.05***		***p*** < ***0.05***		***p*** < ***0.05***	

^α ^Models adjusted by age, sex, urban/rural; Archer and Lemeshow GOF test for weighted logistic regressions displayed for each model; * *p* < 0.05; ** *p* < 0.001.

**Table 7:** Adjusted Odds Ratio (OR)^α^ of presenting each type of disability in the Chilean-born, CASEN survey 2006 (weighted sample size 16,130,746) (statistical significant values appear in grey shade in the table).

Variables	Visual		Hearing		Speaking		Physical		Learning		Psychiatric	
	OR	95%CI	OR	95%CI	OR	95%CI	OR	95%CI	OR	95%CI	OR	95%CI
**CAUSES OF DISABILITY**												
Birth disability	1.00	-	1.00	-	1.00	-	1.00	-	1.00	-	1.00	-
Disease	1.47 **	**1.21**–**1.79**	1.04	0.80–1.35	**0.52** *	**0.32**–**0.77**	1.55 *	**1.29**–**1.86**	0.24 **	**0.19**–**0.31**	3.07 **	**2.04**–**4.63**
Accident	0.72 *	**0.54**–**0.95**	1.12	0.83–1.53	0.67	0.32–1.40	4.27 **	**3.41**–**5.34**	0.22 **	**0.16**–**0.35**	1.12	0.63–1.96
Other non stated	2.35 **	**1.89**–**2.92**	**1.44** *	**1.08**–**1.92**	0.67	0.37–1.24	0.49 *	**0.37**–**0.96**	0.16 **	**0.11**–**0.22**	4.46 **	**2.89**–**6.98**
***GOF test***	***p*** > ***0.05***		***p*** < ***0.05***		***p*** < ***0.05***		***p*** > ***0.05***		***p*** < ***0.05***		***p*** < ***0.05***	
**DEMOGRAPHICS**												
**Age**	**1.03** *	**1.02**–**1.04**	**1.05** *	**1.04**–**1.06**	1.01	0.99–1.02	**1.05** *	**1.04**–**1.06**	**1.03** *	**1.02**–**1.04**	**1.02** *	**1.01**–**1.03**
**Sex **(**female** **=** **1**)	**1.22** *	**1.04**–**1.23**	**0.66** *	**0.57**–**0.76**	0.48	0.35–0.68	1.01	0.91–1.11	**0.74** *	**0.63**–**0.87**	1.06	0.82–1.37
**Marital status:**												
**Single**	1.00	-	**1.00**	**-**	1.00	-	1.00	-	1.00	-	1.00	-
Married	0.98	0.87–1.11	0.75 *	**0.61**–**0.91**	**0.39** *	**0.25**–**0.60**	0.57 *	**0.50**–**0.64**	0.07 **	**0.05**–**0.09**	0.31 **	**0.22**–**0.44**
Divorced	1.17	0.95–1.44	0.65 *	**0.46**–**0.93**	0.63	0.24–1.62	0.85	0.69–1.05	0.10 **	**0.06**–**0.17**	1.22	0.76–1.97
Widow	0.87	0.72–1.04	**0.93**	**0.43**–**1.20**	0.98	0.47–2.05	0.64 *	**0.57–0.77**	0.18 **	**0.13**–**0.26**	0.34 *	**0.19**–**0.61**
**Ethnicity: any**	**1.21** *	**1.01**–**1.44**	1.28	0.94–1.74	1.50	0.93–2.44	0	-	1.10	0.84–1.44	0.90	0.55–1.46
**Zone: rural** ** =** ** 1**	**0.81** *	**0.74**–**0.90**	1.10	0.96–1.26	**0.79** *	**0.56**–**0.60**	**1.29** **	**1.18**–**1.41**	**1.38** **	**1.20**–**1.59**	**0.63** **	**0.43**–**0.80**
**Area:**												
Northern	1.00	-	1.00	-	1.00	-	1.00	-	1.00	-	1.00	-
Central	0.90	0.70–1.07	0.84	0.66–1.08	0.67	0.41–1.12	1.23 *	**1.01**–**1.50**	1.24	0.92–1.68	1.55	0.97–2.47
Southern	0.85	0.71–1.01	0.79	0.61–1.01	0.72	0.43–1.21	1.43 *	**1.17**–**1.74**	1.31	0.97–1.76	1.37	0.84–2.22
***GOF test***	***p*** > ***0.05***		***p*** > ***0.05***		***p*** < ***0.05***		***p*** > ***0.05***		***p***< ***0.05***		***p*** < ***0.05***	
**SOCIOECONOMIC FACTORS**												
**Educational level:**												
No education	1.12	0.62–2.04	**4.60** *	**1.22**–**17.2**	7.5 *	**1.94**–**85.4**	0.94	0.35–2.55	23.0 **	**5.74**–**84.75**	0.24	0.03–1.87
Primary School	**1.41** **	**1.22**–**1.97**	2.02	0.94–4.37	3.48 *	**1.23**–**10.5**	1.29	0.57–2.93	8.68 **	**1.88**–**34.00**	0.54	0.11–2.53
High School	1.31	0.96–1.78	1.42	0.69–2.95	1.38 *	**1.08**–**8.89**	0.96	0.44–2.11	6.85	0.96–18.96	0.48	0.12–1.90
Technical level	1.11	0.81–1.52	1.37	0.62–3.02	1.67 *	**1.12**–**2.84**	1.23	0.51–2.96	6.06	0.65–16.20	0.73	0.13–3.93
University level	1.00	-	1.00	-	1.00	-	1.00	-	1.00	-	1.00	-
**Household income:**												
Quintile 1 (poorest)	1.17	0.84–1.63	1.11	0.69–1.78	2.08	0.68–6.38	**2.26** *	**1.18**–**4.34**	0.53	0.21–1.31	0.42	0.11–1.62
Quintile 2	1.26	0.97–1.64	0.79	0.49–1.26	1.30	0.44–3.82	1.28	0.72–2.28	0.35 *	**0.13**–**0.75**	1.05	0.32–3.23
Quintile 3	1.24	0.96–1.62	1.02	0.62–1.68	0.50	0.16–1.55	1.58	0.39–2.79	0.22 *	**0.07**–**0.67**	1.72	0.49–5.93
Quintile 4	1.12	0.88–1.43	1.20	0.76–1.88	0.57	0.18–1.78	1.26	0.76–2.11	0.31 *	**0.13**–**0.74**	0.94	0.34–2.56
Quintile 5 (wealthiest)	1.00	-	1.00	-	1.00	-	1.00	-	1.00	-	1.00	-
Currently worker	0.81	0.65–1.02	**0.68** *	**0.48**–**0.96**	0.45	0.15–1.33	0.88	0.55–1.19	0.34	0.18–1.61	**0.46** *	**0.22**–**0.97**
***GOF test***	***p***> ***0.05***		***p*** < ***0.05***		***p*** < ***0.05***		***p*** > ***0.05***		***p*** < ***0.05***		***p*** < ***0.05***	
**MATERIAL FACTORS**												
**Quality of the household:**												
Acceptable	1.00	-	1.00	-	1.00	-	1.00	-	1.00	-	1.00	-
Sub-standard	0.95	0.78–1.14	1.01	0.81–1.26	1.47	0.81–2.65	1.16	0.99–1.36	0.91	0.71–1.12	0.71	0.48–1.07
Unfit	1.09	0.79–1.73	0.67	0.38–1.18	1.90	0.41–8.75	1.16	0.80–1.69	1.47	0.86–2.49	0.30	0.10–0.87
Sanitary Index (deficient = 0)	1.28	0.98–1.67	1.23	0.96–1.68	1.10	0.60–2.02	1.01	0.82–1.22	1.56	1.14–2.15	1.63	0.97–2.74
Overcrowded household (Townsend):	0.92	0.78–1.08	0.99	0.82–1.09	0.96	0.71–1.30	0.89	0.76–1.04	0.91	0.71–1.04	**0.65** *	**0.46**–**0.91**
HAI	1.87	0.78–4.70	1.73	0.16–5.16	1.35	0.16–4.38	1.21	0.50–2.50	**2.79** *	**1.11**–**7.00**	6.52	0.95–44.25
***GOF test***	***p*** > ***0.05***		***p*** < ***0.05***		***p*** < ***0.05***		***p*** > ***0.05***		***p*** < ***0.05***		***p*** < ***0.05***	

^α ^Models adjusted by age, sex, urban/rural; Archer and Lemeshow GOF test for weighted logistic regressions displayed for each model; * *p* < 0.05; ** *p* < 0.001.

**Table 8:** Summary of key patterns of factors associated to disability among international immigrants and the Chilean-born, CASEN survey 2006 *.

	Visual	Hearing	Speaking	Physical	Learning	Psychiatric
**Factors associated with among immigrants in Chile, by type of disability:**						
***Crude Rate*** (***%***)	***1.00***	***0.59***	***0.19***	***0.38***	***0.23***	***0.21***
Cause of disability						
Demographic						
SES						
Material						
Migration-related factors						
**Factors associated with disability in the Chilean-born, by type of disability:**						
***Crude Rate*** (***%***)	***3.17***	***1.22***	***0.32***	***2.15***	***0.86***	***0.41***
Cause of disability						
Demographic						
SES						
Material						

* Partially adjusted models (by demographics), a tick represents that at least one variable of each set of factors is significantly associated to the health outcome. The tick represents a statistical significant association at a 5% level.

## 4. Discussion and Conclusions

This is the first study exploring the relationship between disability and SES in the international immigrant population in Chile. Descriptive findings of the prevalence of any and each type of disability were consistent with those reported in the past in the total population in Chile [69]. This is, however, the first population-based study of the prevalence of disability in the immigrant population and exploration of the relationship between disability and SES in this group, so there are not previous studies to compare these results to. National surveys in Chile have shown an increase in the rate of long-term conditions like disability over time, especially affecting those living in the lower SES strata and this was also found in this study, among both the immigrant population and the Chilean-born.

As a summary of key findings from this study, international immigrants reported a lower prevalence of any disability and the six different types of disability included in the CASEN survey. Despite no clear gradients being found in terms of length of stay in the country, there was a significant three times higher chance of having any disability when living for 20 years or more in Chile. Visual and hearing disability types were the most prevalent types among immigrants, while visual and physical were the most frequently reported among the Chilean-born. When observing differences in any disability by age groups, classic measures of SES (income, education and occupation) were independently and consistently associated with any disability in the Chilean-born, whereas latent cluster groups combining such measures together became a better representation of SES in most of the models estimated among the international immigrant population. In addition to this, each type of disability showed a distinctive pattern of related social determinants of health in the two populations under study. In this respect, there is no simple or single pattern of factors associated to the different types of disability between the populations under study. 

International evidence has found that income, education, occupation and material living standards have a gradient of disability across different domains of functionality [91,92,93,94,95]. This is consistent with the key findings from the present study. In addition, many other studies have analysed a particular type of disability or the relationship to disability alone [96,97,98,99]. This study informs current evidence through a broader understanding of a wide range of types of disabilities and their association with different sets of sociodemographic factors.

This study is not free of methodological challenges. First, the data used in our study belonged to a large national representative survey from Chile. Due to the cross-sectional nature of this study, we cannot determine whether migration is a cause of poor SES or disability [100]. Nonetheless, the discussion on the causal relationship between migration, SES and health has been considered extensively in the past decades, including through longitudinal analysis, suggesting a strong link between them [101,102,103,104,105]. We cannot conclude the direction of causal association between SES and disability in this study. However, a clear inverse gradient was found in many of the types of disability analysed, being more prevalent when living in lower SES. These findings are useful to raise hypotheses on plausible causal links between them, which could be further explored with longitudinal datasets when they become available in Chile and the Latin American region. Second, and similar to previous point, we cannot conclude that length of stay in the country is a risk factor for disability in immigrants, but a significant association between any disability and over 20 years spent in Chile was observed. While this may be primarily related to age (cohort effect), it may also represent a worsening of health of immigrants with increased time in Chile due to acculturation processes and should receive further exploration. Third, there is also the risk of self-report bias in this study, not only on migration status, but also SES and disability. Although some limitations of these measures have been recognized in the past, they are considered robust measures and are widely used in health research [106]. Fourth, this study examined some rare events and interpretation of such estimates should be done cautiously. For example, the prevalence of speaking disability was below 0.19% among immigrants and hence the lack of significance for some associations estimated from analysis might be due to lack of statistical power. The over-broad confidence intervals observed for some coefficients also suggests a potential risk of unstable estimation for immigrants, which should be tested in future, ideally prospectively. 

Overall, results in this study showed there is no simple story to tell about disability among immigrants in Chile. Multiple complex patterns are present and similarities and discrepancies between immigrants and the Chilean-born may mask different explanatory mechanisms that are hard to disentangle in a single cross-sectional survey. Despite these limitations, findings from this study contribute to current understanding of disability among international immigrants in Chile, the region and more generally. Most models estimated in this study indicated adequate goodness of fit and hence the interpretation of its results should be considered to inform both policy and health researchers in related fields.

This study reflects the complexity involved in using a multiple sets of factors (*i.e.*, social determinants of health) simultaneously in analysis. Both disability and SES are multidimensional latent social constructs that pose great challenges on their measurement and interpretation [107,108,109]. For example, the need to consider the upper-level social and environmental context [110,111] and a life course approach when defining and measuring these factors should be further explored in Chile, Latin America and more generally. Besides, factors like stress, social support, trust, self-perception of occupational control and autonomy, among others, should be considered in future studies on the health of international immigrants in Chile.

To conclude, this study indicates that international immigrants living in Chile showed lower rates of any disability and six different types of disability compared to the Chilean-born. Although no clear gradients were found in terms of length of stay in the country, immigrants had a significant three times higher chance of having any disability when living for 20 years or more in Chile. Both populations reported visual disability as the most frequent one, but hearing disability was the second most prevalent among immigrants and physical the second most prevalent among the Chilean-born. When stratifying by age groups, some differences in the patterns of factors associated with any disability appeared, but SES remained consistently associated with this outcome. In other words, despite there being different stories to tell regarding factors associated with disability between the immigrants and the Chilean-born, and between different types of disabilities, SES remained a key determinant. In this regard, the lower the SES, the higher the chance of disability in most age groups. These findings suggest the complex relationship between socioeconomic status and material living conditions in developing countries like Chile and should be further explored, particularly when looking at heterogeneous migrant populations. In contrast, when comparing the factors associated to each of the six types of disability between the immigrant and the Chilean-born, no evident similarities appeared and different patterns of sociodemographic factors emerged between the populations under study. These findings suggest that explanatory mechanisms for each type of disability might be different between immigrants and the Chilean-born, and therefore further more detailed analysis should be developed. This could include, besides demographic characteristics, SES, material factors and migration-related factors; reasons for migration, behaviours, perceived stigma and discrimination, access and use of the Chilean healthcare system, and potential protective factors like social cohesion, social capital, and migration-related supportive social networks.
